# Dynamic transfer learning with co-occurrence-guided multi-source fusion for urban spatio-temporal crime prediction

**DOI:** 10.3389/fdata.2026.1697392

**Published:** 2026-02-05

**Authors:** Chen Cui, Ziwan Zheng, Hao Du, Wen Wang

**Affiliations:** 1 Key Laboratory of Public Security Information Application Based on Big-data Architecture, Ministry of Public Security, Hangzhou, China; 2 College of Computer Science and Technology, Hangzhou Dianzi University, Hangzhou, China; 3 Zhejiang SUPCON Information Co., Ltd, Hangzhou, China

**Keywords:** adaptive weight updating, co-occurrence phenomenon of crimes, spatio-temporal crime prediction, transfer learning, urban crime

## Abstract

Spatio-temporal crime prediction is crucial for optimizing police resource allocation but faces challenges including data sparsity, which hinders models from extracting effective patterns and limits robustness—and the underutilization of cross-type crime co-occurrence correlations. To address these issues, we propose a transfer learning approach that explores underlying cross-type relationships, enabling the sharing of spatio-temporal features across crime types and alleviating data sparsity. An adaptive weight updating mechanism is incorporated to enhance the perception of distinct crime categories, while the impacts of points of interest (POIs), meteorological factors, and other features are also analyzed. Experiments on real-world data from a Chinese city show that our model comprehensively captures latent features across crime types, thereby enhancing predictive performance and robustness, particularly for crime types with sparse data. Moreover, it effectively incorporates environmental features, further improving crime prediction performance.

## 1 Introduction

Spatio-temporal crime prediction holds great significance, as it can provide guidance for police resource allocation, thereby reducing public property losses. It is pivotal for enabling proactive policing, optimizing resource efficiency, and enhancing urban safety management—especially in densely populated urban areas where crime incidents are diverse and dynamic, placing high demands on real-time and precise security deployment. With the acceleration of urbanization and the expansion of urban areas, the complexity of crime patterns has increased, making traditional reactive policing strategies inadequate. Thus, this topic increasingly attracts the attention of researchers. Traditional spatio-temporal crime prediction is primarily based on either one or both of the temporal and spatial correlations in crime (Zhao and Tang, 2017), with representative approaches including crime near-repeat models (Townsley et al., 2003), kernel density estimation models (Bowers et al., 2004; Kalinic and Krisp, 2018), and self-exciting point process models (D'Orsogna and Perc, 2015; Mohler et al., 2011). However, these traditional approaches exhibit limited applicability. On the one hand, these models are too simplistic, making it challenging to capture complex spatio-temporal correlations in crime. On the other hand, these traditional approaches do not take into account multiple crime-related auxiliary data sources such as points of interest (POIs) and weather data.

With the increasing application of AI technology, research in geospatial artificial intelligence (GeoAI) has been on the rise. GeoAI models can capture complex spatio-temporal correlations and extract features from multiple auxiliary data. Currently, they are mainly applied to spatio-temporal prediction tasks in data-intensive domains such as traffic flow, temperature, and air quality (Gao, 2021; Deng et al., 2025). These methods have achieved favorable predictive results, prompting researchers to actively study spatio-temporal crime prediction using these techniques in recent years (Sun et al., 2023). However, several challenges persist in the field of spatio-temporal crime prediction.

### 1.1 Challenge 1 (addressing the sparsity of spatio-temporal crime data)

Previous research has demonstrated that crime is not evenly distributed in space, and crime data are sparse and not continuous in both time and space dimensions (Andresen and Malleson, 2011; Chainey and Ratcliffe, 2013; Ratcliffe, 2004), making spatio-temporal crime prediction a challenging task (Weisburd, 2015). A common strategy to address sparsity is spatiotemporal aggregation, which increases data density by predicting for larger areas or longer periods. However, this leads to a coarsening of analytical granularity, sacrificing detailed insights for statistical stability. To address this issue, current methods have focused on densifying crime data. Typically, smoothing techniques are employed to create a pseudo-continuity in the temporal dimension (Calatayud et al., 2023; Kumar et al., 2018; Taddy, 2010; Zhang and Cheng, 2020). When dealing with spatial dimensions, it is common to apply weighted smoothing techniques that account for the near-repeat effect (Calatayud et al., 2023; Hart et al., 2022; Johnson et al., 2009; Kidner et al., 2002). However, the potential problems and limitations of using smoothing techniques to handle sparse data, such as information loss and overfitting, have affected the performance of spatio-temporal crime prediction models. In summary, it is both important and challenging to address the sparsity in spatio-temporal crime prediction.

### 1.2 Challenge 2 (modeling cross-type temporal–spatial correlation adequately)

A previous study (Zhao et al., 2022) verified the existence of correlations among different types of crime from temporal and spatial perspectives, that is, the co-occurrence phenomenon among different types of crime (Liu et al., 2018b). These existing research results provide the foundation for leveraging cross-type correlations for accurate spatio-temporal crime prediction, which is often overlooked in most current spatio-temporal crime prediction models. Utilizing these correlations allows models to share learned patterns across crime types, which is particularly beneficial for improving predictions when data for a specific type is sparse.

To address these challenges, based on transfer learning, which aims to leverage knowledge obtained from one domain to enhance learning performance in another, we studied an adaptive transfer learning model for spatio-temporal crime prediction. Specifically, transfer learning is employed to tackle data sparsity by sharing features across crime types, and an adaptive weight mechanism is designed to model cross-type correlations adequately. The following summarizes our main contributions.

(1) Based on the crime co-occurrence phenomenon, a spatio-temporal crime prediction model is proposed using transfer learning, which analyzes spatial, environmental, and temporal characteristics of different types of crimes.
(2) An adaptive method utilized in the transfer learning process allows the model to pay sufficient attention to the influence of different types of crimes, effectively alleviating data sparsity and enhancing the model's robustness and performance for crime types with limited data.
(3) We conducted comprehensive experiments using real-world datasets to assess the performance of the proposed spatio-temporal crime prediction model and analyze the role of each component in the model for crime prediction.

## 2 Related work

In this section, we mainly discuss related work on spatio-temporal crime prediction based on artificial intelligence.

### 2.1 Traditional machine learning models

The random forest, a traditional machine learning model, has been widely applied in predicting crime hotspots (Alves et al., 2018). For example, Liu et al. (2018a) adopted the random forest and kernel density method to predict crime hotspot situations across different time periods. However, it focused on the distribution of crime data and ignored the potential influence of spatio-temporal factors such as weather conditions and the built environment on criminal behavior. Therefore, in their subsequent research (Liu et al., 2019), Liu et al. deeply analyzed the spatial variations of crime and the built environment, as well as the varying relationship between crimes and the built environment, employing the random forest algorithm to predict public property crime. This demonstrated that crime prediction models can be improved by incorporating the aforementioned spatial variations and spatially varying relationships.

As another typical machine learning model, boosting learning algorithms have also been widely applied to address crime prediction challenges. For example, Zhang et al. (2022) and Deng et al. (2023) compared the XGBoost model with other popular machine learning models such as logistic regression, decision trees, and random forests; the XGBoost model clearly showed the best fit. Kim and Lee (2023) also stated that the Light Gradient Boosting Machine model (LightGBM) was chosen as the most suitable model for predicting crime incidence in 250 m grid units of Seoul by comparing linear regression, random forests, and multi-layer perceptrons.

Although random forests and boosting learning algorithms have shown promising results in crime prediction, their relatively lower model complexity may limit their ability to capture highly intricate non-linear spatio-temporal relationships. Additionally, when dealing with extensive and diverse crime data and multiple crime-related auxiliary data sources, they might encounter constraints in terms of their robustness, potentially overfitting or failing to capture underlying relationships within the data.

### 2.2 Deep learning models

Deep learning models stand out for their capability to automatically learn hierarchical feature representations directly from raw, high-dimensional spatio-temporal data, an advantage over many machine learning methods that often rely on carefully engineered features. This representation learning capability, coupled with their ability to model complex non-linear relationships, further advances their adoption in this field. For example, Yan and Hou (2020) employed a long short-term memory (LSTM) neural network, which is particularly adept at capturing temporal dependencies, for theft crime prediction. Wu et al. (2023) researched the issue of fairness in crime prediction models implemented with deep learning approaches. Gao et al. (2022) combined LSTM and regression to predict telecommunication network fraud crimes. In addition, due to their inductive bias for spatial structures, convolutional neural networks (CNNs) and graph neural networks (GNNs) have been widely applied in spatio-temporal crime prediction. For example, Duan et al. (2017) utilized a deep convolutional neural network for spatio-temporal crime prediction. Graph-based models were proposed to capture dynamic patterns of criminal behavior for crime prediction (Sun et al., 2023; Han et al., 2020; Wang et al., 2020, 2018). Recently, Shahmoradi et al. (2025) proposed a hybrid model integrating ST-ResNet and LSTM for precise crime hotspot prediction, demonstrating the effectiveness of combining spatial and temporal deep learning architectures. Overall, researchers are currently primarily focused on spatio-temporal crime prediction based on deep learning. However, there are not many research results on the issues of the sparsity of spatio-temporal crime data and the cross-type correlations of different types of crime so far, requiring further investigation. Specifically, the potential of explicitly modeling cross-type correlations for knowledge transfer and as a built-in mechanism to counteract data sparsity remains underexplored. Here, transfer learning, which has been widely applied in fields such as computer vision (Gopalakrishnan et al., 2017; Shin et al., 2016), natural language processing (Ruder et al., 2019), and autonomous driving (Huang et al., 2018), is adopted for spatio-temporal crime prediction to effectively utilize knowledge transfer to learn the relationships between crime types and alleviate the problem of data sparsity for certain crime types.

## 3 Preliminaries

**Urban Data:** The information on crime occurrences, including crime type, timestamp, latitude, longitude, and surrounding environmental conditions, is recorded. Every crime report is mapped to a geographic region based on its location. Here, we begin with some necessary notations and then formally present the problem studied in this work. Particularly, we consider a set of *R* regions in a city, *K* types of crime (e.g., burglary and assault), and a sliding window with *T* time slots (e.g., days). Define *r, i*, and *t* as the indices for the region, crime type, and time slot, respectively. Let ${\text{X}}_{\text{i}}\in {\mathbb{R}}^{R\times T\times \left(1+H\right)}$ denote the observed *i*-th type of crime and crime-related auxiliary data source, where the elements *X*_*i, r, t*, 1_ and *X*_*i, r, t, h*_ are, respectively, the number of *i*-th type of crime and the *h*-th crime-related auxiliary feature (e.g., temperature and rainfall) observed at the *r*-th region in the *t*-th time slot in a sliding window, and *H* is the number of crime-related auxiliary features selected. In addition, define a crime vector ${\text{y}}_{i}\in {\mathbb{R}}^{R}$to represent quantitative distribution of the *i*-th type of crime across regions. Specifically, each element *y*_*i*_(*r*) is the number of *i*-th type of crime at the *r*-th region in the (*T*+1)-th time slot. Furthermore, the supervised processed data can be formulated as $D=\left[{\text{D}}_{1},{\text{D}}_{2}\ldots {\text{D}}_{K}\right]$, where ${\text{D}}_{i}=\{X_{i}^{(n)},y_{i}^{(n)}){\}}_{n=1}^{N}$ and *N* denotes the samples for the *i*-th crime type generated by the sliding window. Here, $(X_{i}^{\left(n\right)},y_{i}^{\left(n\right)})$ denotes the *n*-th sample pair, with $X_{i}^{\left(n\right)}$ being the input tensor and $y_{i}^{\left(n\right)}$ the corresponding output vector for that sample.

**Task Formalization:** The main task is to construct a predictive model for each type of crime that utilizes transfer learning to fully learn the cross-type spatio-temporal correlation characteristics of *K* types of crime. Specifically, the model input is the historical crime counts and feature tensor D, and the output is the predicted crime count—that is, the quantitative distribution of crimes for a certain type at time slot *T*+1. The prediction process can be described as $M:D\to y_{i}\in {\mathbb{R}}^{R}$, where each element of *y*_*i*_ is the predicted crime count of the *i*-th type in the corresponding region. This is a regression task. We aim to predict continuous crime count values, rather than discrete categories.

## 4 Proposed method

**Figure 1:**
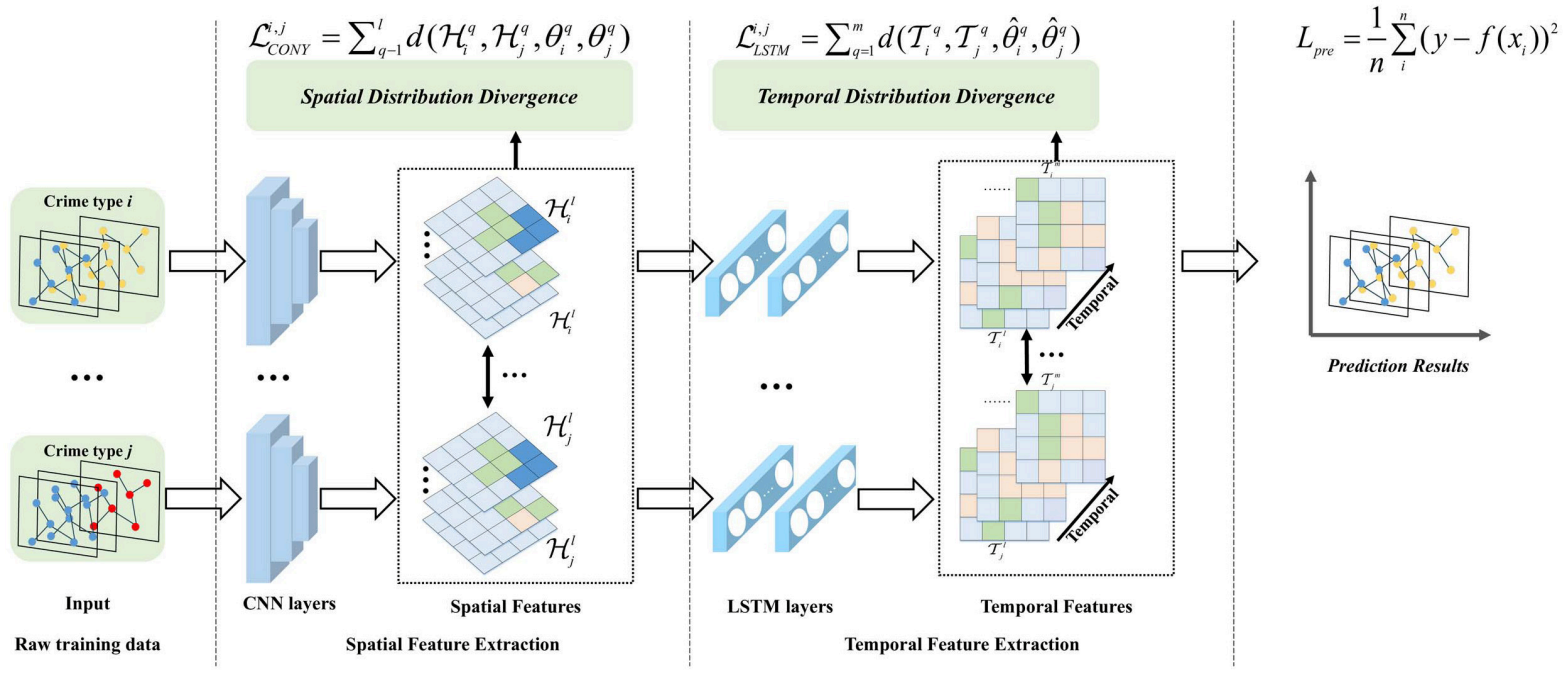
Architecture of proposed model.

The proposed framework—the co-occurrence-guided adaptive transfer learning (CATL) model—based on the co-occurrence phenomenon of crimes, is designed to solve the above-formulated spatio-temporal crime prediction task, as shown in Figure 1. The proposed model consists of two major modules, namely feature extraction and adaptive model optimization. Specifically, we utilize two types of modules to calculate spatial similarity, compute temporal similarity via the temporal feature extraction module, and finally output the crime prediction results. The two modules are explained in detail in the following subsections.

### 4.1 Feature extraction module

First, we construct K independent convolutional long short-term memory [ConvLSTM (Khosravinia et al., 2023)] networks to extract spatial and temporal features of each type of crime. ConvLSTM is a spatio-temporal extension of standard LSTM, integrating convolutional operations into input-to-state and state-to-state transitions. It is selected for our grid-structured crime data because it simultaneously captures spatial correlations via convolutions and temporal dependencies via LSTM gates—avoiding information loss from separate spatial–temporal processing, which is a limitation of other hybrid models. The model architecture, optimized via full grid search, is detailed as follows: convolutional part (1,024 nodes per layer, 3 × 3 convolutional kernels with stride = 1 and padding = 1); recurrent part (1,024 nodes per layer); activation functions (sigmoid for gate control, tanh for state update); and output feature dimensions (spatial *v* = 512, temporal *V* = 512). The number of hidden layers for the convolutional part is *l* = 2 and *that* for the recurrent part is *m* = 2. Let the current input ${\text{X}}_{\text{i}}\in {\mathbb{R}}^{R\times T\times \left(1+H\right)}$ be a segment of data for the *i*−*th* type of crime. In spatial feature extraction, the output ${H=\left\{H_{1}^{q},...,H_{K}^{q}\right\}}_{q=1}^{l}\in {\mathbb{R}}^{K\times l\times v}$ of the K CNNs with *l* hidden layers can be formulated as


$$\begin{array}{l} H^{\text{q}}=\text{Conv}\left(H_{\text{i}}^{\text{q}-1};\theta_{\text{i}}^{\text{q}}\right) \end{array}$$ (1)

$$\begin{array}{l} H_{\text{i}}^{0}=X_{\text{i}}\;L_{\text{CONV}}^{\text{i},\text{j}}L_{\text{LSTM}}^{\text{i},\text{j}} \end{array}$$ (2)

where *v* is the output feature dimension of each hidden layer, *q* represents the *q*−*th* hidden layer, and θ denotes the learnable model parameters of the CNN.

Then, LSTM is used to extract temporal features, and the output ${T=\left\{T_{1}^{q},...,T_{K}^{q}\right\}}_{q=1}^{m}\in {\mathbb{R}}^{K\times m\times V}$ of the *K* LSTMs with *m* hidden layers can be formulated as


$$\begin{array}{l} T_{\text{i}}^{\text{q}}=LSTM\left(T_{\text{i}}^{\text{q}-1};{\hat{\theta }}_{\text{i}}^{\text{q}}\right) \end{array}$$ (3)

$$\begin{array}{l} T_{\text{i}}^{0}=H_{\text{i}}^{\text{l}} \end{array}$$ (4)

where *V* is the feature dimension of each hidden layer and *q* and $\hat{\theta }$ denote the *q*−*th* hidden layer and learnable model parameters of the LSTM, respectively.

After obtaining the output results for each hidden layer of both CNN and LSTM, we need to compute the overall probability distribution differences among crime types. Given that each hidden layer of CNN and LSTM contains only partial information of the input data, all hidden layer outputs should be considered when calculating the overall distribution differences. Given a crime type-pair (**D**_*i*_, **D**_*j*_), the loss of probability distribution differences can be formulated as


$$\begin{array}{l} \left(H_{\text{i}},H_{\text{j}};\theta_{\text{i}},\theta_{\text{j}}\right)=\overset{l}{\underset{q=1}{\sum }}\text{d}\left(H_{\text{i}}^{\text{q}},H_{\text{j}}^{\text{q}};\theta_{\text{i}}^{\text{q}},\theta_{\text{j}}^{\text{q}}\right),1\leq i\neq j\leq K\; \end{array}$$ (5)

$$\begin{array}{l} \left(T_{\text{i}},T_{\text{j}};{\hat{\theta }}_{\text{i}},{\hat{\theta }}_{\text{j}}\right)=\overset{m}{\underset{q=1}{\sum }}\text{d}\left(T_{\text{i}}^{\text{q}},T_{\text{j}}^{\text{q}};{\hat{\theta }}_{\text{i}}^{\text{q}},{\hat{\theta }}_{\text{j}}^{\text{q}}\right),1\leq i\neq j\leq K\; \end{array}$$ (6)

where *d*(·, ·) represents the probability distribution distance function. Considering the sparsity of crime spatio-temporal data, we adopt the maximum mean discrepancy (MMD) as the distance measure *d*(·, ·) to better measure distribution discrepancy among different types of crimes and enhance computational efficiency:


$$\begin{array}{l} {MMD\left(N_{s},M_{t}\right)}^{2}=||\frac{1}{N_{S}}\overset{N_{s}}{\underset{i=1}{\sum }}\varphi \left(x_{i}\right)-\frac{1}{M_{t}}\overset{M_{t}}{\underset{i=1}{\sum }}\varphi \left(y_{j}\right){||}^{2}\\ \;=\frac{1}{{N_{s}}^{2}}\overset{N_{s}}{\underset{i=1}{\sum }}\overset{N_{s}}{\underset{i^{\prime }=1}{\sum }}k\left(x_{i},x_{i^{\prime }}\right)-\frac{2}{N_{s}M_{t}}\overset{N_{s}}{\underset{i=1}{\sum }}\overset{M_{t}}{\underset{j=1}{\sum }}k\left(x_{i},y_{i}\right)\\ \;+\frac{1}{{M_{t}}^{2}}\overset{M_{t}}{\underset{j=1}{\sum }}\overset{M_{t}}{\underset{j^{\prime }=1}{\sum }}k\left(y_{j},y_{j^{\prime }}\right) \end{array}$$ (7)

where *k*(·, ·) is a Gaussian kernel function and *N*_*s*_ = |*N*_*s*_| and *M*_*t*_ = |*M*_*t*_| are the numbers of data points from the respective distributions. Furthermore, considering the particularity of crime data, it is essential for the model to effectively capture low-frequency but high-impact crime patterns. Therefore, we adopt a more sensitive bandwidth function *h* for the Gaussian kernel function *k*(·, ·) as follows:


$$\begin{array}{l} \text{L}=|{\text{N}}_{\text{s}}+{\text{M}}_{\text{t}}|\times \left(|{\text{N}}_{\text{s}}+{\text{M}}_{\text{t}}|-1\right) \end{array}$$ (8)

$$\begin{array}{lll} \text{h} & = & \frac{\sigma }{\text{L}}\sum {||{\text{x}}_{\text{i}}-{\text{x}}_{\text{j}}||}^{2}+\epsilon \;,\;{\text{x}}_{\text{i}},{\text{x}}_{\text{j}}\in {\text{N}}_{\text{s}},{\text{M}}_{\text{t}} \end{array}$$ (9)

where σ is a constant used to control the size of the bandwidth function and ϵ is a small value to prevent numerical instability during computation.

### 4.2 Adaptive model optimization

To minimize the predictive error for each type of crime, the mean square error (MSE) loss function is often adopted.


$$\begin{array}{l} L_{\text{pred}}\left(\theta ,\hat{\theta }\right)=\frac{1}{\left|{\text{D}}_{\text{j}}\right|}\overset{|{\text{D}}_{\text{j}}|}{\underset{\text{n}=1}{\sum }}MSE\left({\text{y}}_{\text{i}}^{\left(\text{n}\right)},M\left(D^{\left(\text{n}\right)};\theta ,\hat{\theta }\right)\right) \end{array}$$ (10)

where |**D**_*j*_| is the total length of the *j*-th type of crime data after being processed with a sliding window.

However, solely minimizing Equation 10 is insufficient for the model to learn the distributional differences among different types of crimes. Therefore, we combine Equation 5 and Equation 6 and introduce distributional discrepancy:


$$\begin{array}{lll} L_{\text{Distribution}}\left({\text{D}}_{\text{i}},{\text{D}}_{\text{j}}\right) & = & L_{\text{CONV}}^{\text{i},\text{j}}\left(H_{\text{i}},H_{\text{j}};\theta_{\text{i}},\theta_{\text{j}}\right)\\ \;+L_{\text{LSTM}}^{\text{i},\text{j}}\left(T_{\text{i}},T_{\text{j}};{\hat{\theta }}_{\text{i}},{\hat{\theta }}_{\text{j}}\right) \end{array}$$ (11)

During the training process, to avoid overly focusing on a few types of crime distributions while neglecting others, we introduce $\alpha \in {\mathbb{R}}^{\frac{K}{2}\left(K-1\right)}$ to update the importance of different types of crime distributions. Here, ${\text{g}}_{\text{i},\text{j}}^{\text{N}+1}$ denotes the rate of change of distribution discrepancy loss between crime types **D**_**i**_ and **D**_**j**_ in the *N*-th epoch. In summary, the loss function and the adaptive weight update rule can be formulated as


$$\begin{array}{l} L\left(\theta ,\hat{\theta },\alpha \right)\\ \;=L_{\text{pred}}\left(\theta ,\hat{\theta }\right)+\lambda \sum_{\text{i}=1}^{\text{K}}\sum_{\text{j}=1,\text{j}\neq \text{i}}^{\text{k}}\alpha_{\text{i},\text{j}}L_{\text{Distributio}\text{n}}\left({\text{D}}_{\text{i}},{\text{D}}_{\text{j}}\right) \end{array}$$ (12)

$$\begin{array}{ll} \alpha_{\text{i},\text{j}}^{\text{N}+1} & =\{\begin{array}{l} \alpha_{\text{i},\text{j}}^{\text{N}}\left(1+\text{s}igmo\text{d}\left({\text{g}}_{\text{i},\text{j}}^{\text{N}+1}\right)\right),\;{\text{g}}_{\text{i},\text{j}}^{\text{N}+1}\geq 0\\ \alpha_{\text{i},\text{j}}^{\text{N}},\;\text{otherwise} \end{array} \end{array}$$ (13)

$$\begin{array}{ll} \alpha_{\text{i},\text{j}}^{\text{N}+1} & =\frac{\alpha_{\text{i},\text{j}}^{\text{N}+1}}{\sum_{\text{j}=1}^{\text{V}}\alpha_{\text{i},\text{j}}^{\text{N}+1}} \end{array}$$ (14)

When $L_{Distribution}\left({\text{D}}_{i},{\text{D}}_{j}\right)$ is greater than epoch *N*, the distribution discrepancy between **D**_*i*_ and **D**_*j*_ has increased, and in epoch *N*+1, we need to increase the importance of this discrepancy by increasing $\alpha_{i,j}^{N+1}$. Finally, we need to normalize the weights.

## 5 Experiments

Here, extensive experiments with real-world datasets from a prefecture-level city in China, are conducted to evaluate the effectiveness of the proposed CATL model. We mainly aim to answer three questions:

(1) How do the cross-type spatio-temporal correlations and the adaptive methods adopted in the transfer learning process benefit spatio-temporal crime prediction?
(2) What is the performance of variants of the proposed spatio-temporal crime prediction model with different combinations of crime-related auxiliary data?
(3) How does the proposed CATL model perform compared to state-of-the-art baselines?

### 5.1 Settings

#### 5.1.1 Dataset description

In accordance with data security and privacy protocols, the source province is referred to anonymously as “J Province” in this paper. The experiment was conducted in a prefecture level city in J Province, China, which covers an area of 668 km^2^ and has a registered resident population of 714,000. Some sparsely populated areas in the city, such as villages and farmland, have relatively low crime rates and hold little significance for spatio-temporal crime prediction. Hence, the central urban area of the city was chosen as the research area. The selected area covers 243 km^2^, accounting for 30.8% of the registered residential population. We collected five types of crime data (i.e., burglary, assault, rape, drugs, and gambling) from January 2014 to August 2021, totaling 32,531 records. The sparsity level of each crime type—quantified by average records per 2 *km* × 2 *km* grid per day—is as follows: burglary (0.12), assault (0.0035), drugs (0.0062), gambling (0.0025), and rape (0.0013), showing varying degrees of sparsity consistent with spatio-temporal granularity constraints. This data was pre-processed through a standard spatio-temporal data processing pipeline: each record was mapped to a 2 *km* × 2 *km* grid and aggregated into daily time slots to form the model's input tensors. To ensure privacy, all personal identifiers were removed, and precise locations and timestamps were obfuscated through spatial aggregation and daily temporal aggregation, respectively. This selection was based on data availability and operational definitions from our data source; we note that the data reflect reported incidents. The processed data were integrated into structured tensors compatible with the model input requirements. The crime-related environmental features include weather, POI, and other features such as historical surveillance count (the number of registered public security cameras in the target grid during the corresponding time slot) and population count, as detailed in Table 1.

**Table 1:** Crime-related auxiliary features.

**Category**	**Variable**	**Description**
Weather features	Temperature	Temperature variance, maximum, and minimum values in time slots
	Atmospheric pressure	Atmospheric pressure variance, maximum, and minimum values in time slots
	Humidity	Humidity variance, maximum, and minimum values in time slots
	Rainfall	Rainfall variance, maximum, and minimum values in time slots
	Wind speed	Wind speed variance, maximum, and minimum values in time slots
POI features	Public sector industry	Public activity places, including KTV, tea houses, large shopping malls, cinemas, video game rooms, bars, beauty salons, chess and card rooms, internet cafes, dance halls, bathhouses, foot massage parlors, and other publicly accessible venues with high human traffic
	Special industry	Places engaged in special industries, including pawn shops, hotels, used car dealerships, real estate agencies, scrap recycling, seal engraving, automotive repair, courier logistics, consignment businesses, jewelry trade, locksmith services, car rental, mobile communications, material distribution, and plate printing
	Ordinary unit	Places that are not considered as the key, public, special, or hazardous units, including ordinary companies, restaurants, factories, fruit and vegetable stalls, department stores, pharmacies, etc.
	Major unit	Places that need special protection by public security authorities, such as stations, urban water supply facilities, government offices, gas stations, gas companies, schools, hospitals, banks, etc.
Other features	Historical surveillance count	Number of surveillance counts in each region in time slots
	Population counts in each region	Population counts in time slots

**Figure 2:**
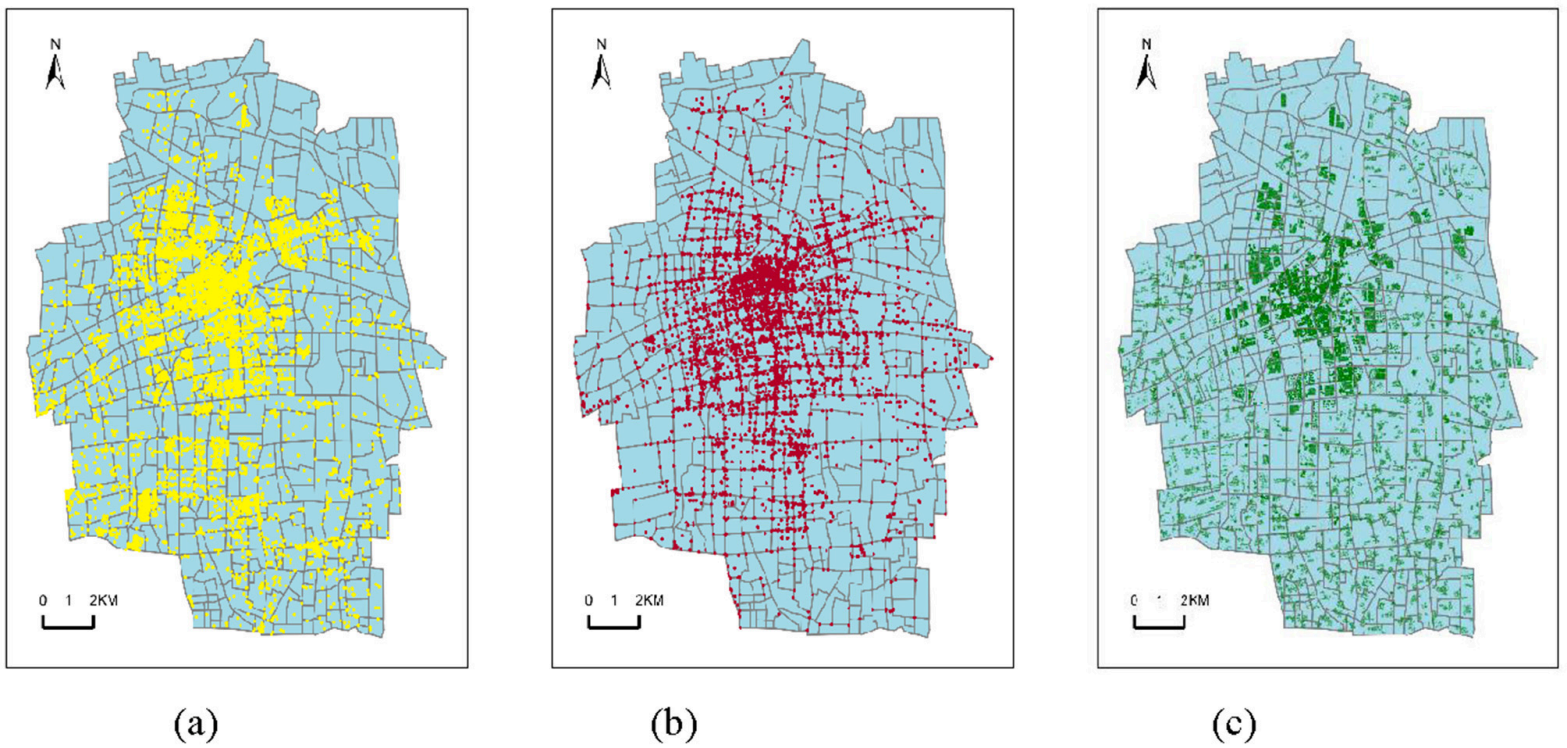
Spatial distributions of different features. **(a)** POI. **(b)** Surveillance. **(c)** Population.

The distributions of POI, surveillance, and population are illustrated in Figure 2.

#### 5.1.2 Experimental setup

The basic experimental setup is summarized as follows:

(1) Applying a 2 *km* × 2 *km* grid unit, we generated a total of 90 spatial regions. We set a 1-day period as the fixed temporal granularity. Crucially, even at this 2 *km* × 2 *km* and daily resolution, the crime event data exhibits substantial sparsity (with many grid-day cells containing zero events), establishing a meaningful and challenging tested for evaluating our model's capability to mitigate sparsity issues. The dataset was divided into training sets, validation sets, and test sets in a 7:1:2 ratio. The split was performed in strict chronological order. Furthermore, a gap equal to the input sequence length (*T*) was introduced between the sets to prevent data leakage caused by the sliding window. This procedure ensures that the sliding windows are constructed independently within each chronologically ordered subset, eliminating any temporal overlap and thus preventing the data leakage scenario of concern.
(2) A full grid search was performed for hyperparameter optimization of the ConvLSTM models. Specifically, for all baseline models and our proposed CATL model, we conducted independent hyperparameter tuning to ensure a fair comparison and avoid experimental bias. For each model, we performed a grid search over key hyperparameters (e.g., number of layers, hidden units, learning rate, batch size, and regularization weight λ) and selected the configuration that yielded the best validation performance through iterative training. A series of comparative experiments yielded the hyperparameter settings. Adam with default parameters was used as the optimizer, and the learning rate was set to 0.001. The spatial feature extraction module and temporal feature extraction module consist of multivariate convolutional layers and LSTM layers. The batch size was selected from the candidate set {10, 12, 14, 16, 18, 20, 22, 24}. The weight for the regularization term λ in Equation 12 was selected from the range of [0.0, 1.0] with a step size of 0.1.
(3)The crime prediction performance is evaluated using mean absolute error (MAE) and mean squared error (MSE), which are formulated as follows:
$$\begin{array}{l} MAE\left(\text{y},\hat{\text{y}}\right)=\frac{1}{\text{n}}\overset{\text{n}}{\underset{\text{i}=1}{\sum }}|{\text{y}}_{\text{i}}-{\hat{\text{y}}}_{\text{i}}| \end{array}$$ (15)
$$\begin{array}{l} MSE\left(\text{y},\hat{\text{y}}\right)=\frac{1}{\text{n}}\overset{\text{n}}{\underset{\text{i}=1}{\sum }}{\left({\text{y}}_{\text{i}}-{\hat{\text{y}}}_{\text{i}}\right)}^{2} \end{array}$$ (16)
  MAE and MSE provide complementary perspectives: MAE reflects the average error magnitude, while MSE is more sensitive to large, sporadic prediction errors—a critical consideration in public safety applications, where *n* is the number of test samples and *y*_*i*_ and ŷ_*i*_ are the predicted and observed crime amounts, respectively.
(4) In the evaluation, the results are the median values obtained after conducting 10 independent experiments. Here, independent experiments refer to using 10 different random seeds to control the randomness of model weight initialization and data division, ensuring the reproducibility of the results. We chose the median instead of the mean because some crime types (e.g., rape and gambling) have extremely sparse data, and occasional outlier values may occur in repeated experiments. The median is more robust to such outliers and can objectively reflect the true performance of the model.

#### 5.1.3 Baselines for comparison

We compared the performance of the proposed CATL model with the following baseline methods.

**TGCN** (Chen et al., 2020): The tag graph convolutional network is a valuable tool for capturing both spatial and temporal dependencies and for understanding and predicting complex temporal graph-structured data. It has shown promising results in various applications. Here, the TGCN module uses two layers and 1,024 hidden nodes.

**DCRNN** (Li et al., 2017): The diffusion convolutional recurrent neural network uses diffusion convolution to model spatial dependencies in the data, taking into account the structural connections between different locations. We chose the number of neighboring nodes K as 2, with 1,024 hidden nodes.

**GRU** (Dey and Salem, 2017): The gated recurrent unit neural network is a high-performing and widely used predictive model with a simple structure, fast training speed, and the ability to effectively capture temporal features.

**LSTM** (Graves, 2012): The long short-term memory neural network is widely used in time-series forecasting, effectively handling long-term dependencies and being suitable for multivariable predictions.

**ConvLSTM** (Khosravinia et al., 2023): Convolutional LSTM can capture spatiotemporal information from the input data while considering spatiotemporal sequence features. The CNN module consists of two layers, and the number of LSTM hidden nodes is set to 1,024.

**GConvLSTM** (Yuan et al., 2018): Graph convolutional LSTM is designed for spatiotemporal data and can integrate spatiotemporal information, handle complex relationships, and efficiently share parameters. In this case, the GCN module has two layers, and the number of LSTM hidden nodes is set to 1,024.

**STGCN** (Yu et al., 2018): The spatio-temporal graph convolutional network is designed for spatiotemporal prediction. It can simultaneously capture temporal and spatial relationships and offers numerous advantages when dealing with intricate spatiotemporal relationships and graph-structured data, including low computational complexity and efficient feature processing.

**PDFormer** (Jiang et al., 2023): Spatio-temporal graph convolutional network is designed for spatiotemporal prediction. It can concurrently capture temporal and spatial relationships and has numerous advantages when dealing with intricate spatiotemporal relationships and graph-structured data, including low computational complexity and efficient feature processing.

### 5.2 Ablation study on transfer learning components

**Figure 3:**
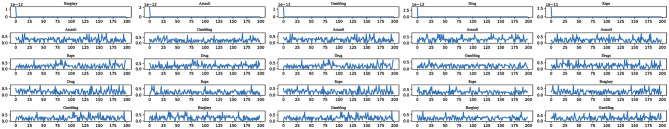
Weight updating of probability distribution loss.

As described in Section 4.2, an adaptive weighting mechanism is incorporated to enable the model to fully learn the probability distributions among different types of crimes. It ensures that the proposed CATL model does not overly prioritize the differences in probability distributions between certain pairs of crime types during the learning process, thereby neglecting relationships with other crime types. Figure 3 illustrates the weight update process, where the *x*-axis represents the training epoch and the *y*-axis denotes the value of the adaptive weight. When predicting a certain type of crime, theoretically, the probability distribution difference for the same type of crime should be 0 during the training process. Thus, the weight for this aspect should eventually approach 0. To validate the correctness of our model, we also incorporate the calculation of the probability distribution difference between the model and itself during the computation process. The observations from Figure 3 confirm this perspective, thereby validating our approach.

**Figure 4:**
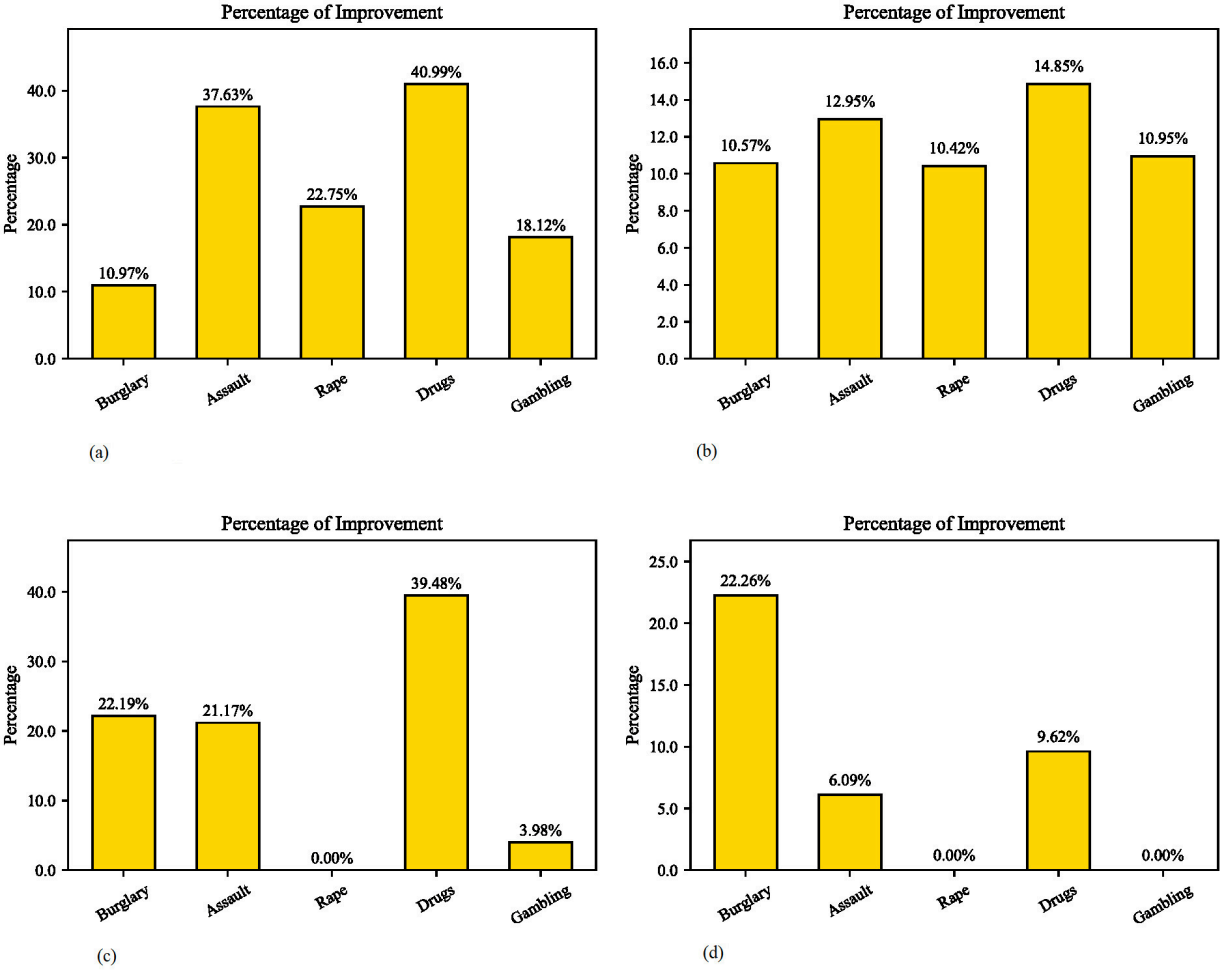
MAE and MSE improvements of our model compared with other two methods. **(A)** MAE improvement between base ConvLSTM and CATL Model. **(B)** MAE improvement between CATL w/o Adaptive and CATL Model. **(C)** MSE improvement between base ConvLSTM and CATL Model. **(D)** MSE improvement between CATL w/o adaptive and CATL model.

Furthermore, to better understand the CATL model and validate its effectiveness, we compared the crime prediction performance of our model without transfer learning (i.e., ConvLSTM, referred to as “BaseConvLSTM” here) and the model without adaptive weight updating (“CATL w/o Adaptive”). Table 2 presents the MAE and MSE of the three compared methods, and Figure 4 shows the improvement in MAE and MSE of our model compared to the other two methods. From the results, two key observations are summarized as follows.

**Table 2:** Performance of different methods.

**Crime category**	**BaseConvLSTM**		**CATL w/o Adaptive**		**CATL Model**	
	**MAE**	**MSE**	**MAE**	**MSE**	**MAE**	**MSE**
Burglary	0.4231	0.8869	0.4212	0.8877	0.3767	0.6901
Assault	0.0388	0.0137	0.0278	0.0115	0.0242	0.0108
Rape	0.0167	0.0093	0.0144	0.0093	0.0129	0.0093
Drugs	0.0544	0.0233	0.0377	0.0156	0.0321	0.0141
Gambling	0.0298	0.0226	0.0274	0.0217	0.0244	0.0217

(1) Our model outperforms the other two models in predicting all types of crimes, and the CATL w/o Adaptive performs better than the BaseConvLSTM. This indicates that there are indeed certain underlying relationships between the probability distributions of burglary, assault, drugs, and other crime types. The model consistently focuses on the differences in probability distributions among various crime types in each epoch through the adaptive method adopted in the transfer learning process, allowing it to effectively capture the mutual influence between crimes. At the same time, it demonstrates that the proposed CATL leverages feature sharing in transfer learning to alleviate the issue of data sparsity.

(2) It can be inferred that our model exhibits heightened sensitivity to the varied distributions associated with burglary, assault, and drug abuse, as evidenced by the significant improvement in both MAE and MSE for these three crime types in Figure 4. This implies that the proposed CATL shows greater robustness in predicting these three types of crimes. On the other hand, there is noticeable improvement in the MAE for rape and gambling, while the MSE remains unchanged or shows little improvement. Figure 4's 0% MSE improvement for rape corresponds to the identical MSE value (0.0093) of all three models in Table 2, indicating no difference in prediction error. A negative percentage would be recorded if our model performed worse. Considering the adoption of median-based metrics and the pronounced sensitivity of MSE to errors, this suggests that the model strives to minimize errors as much as possible during optimization.

### 5.4 Impact of environmental features (weather/POI) on prediction

**Figure 5:**
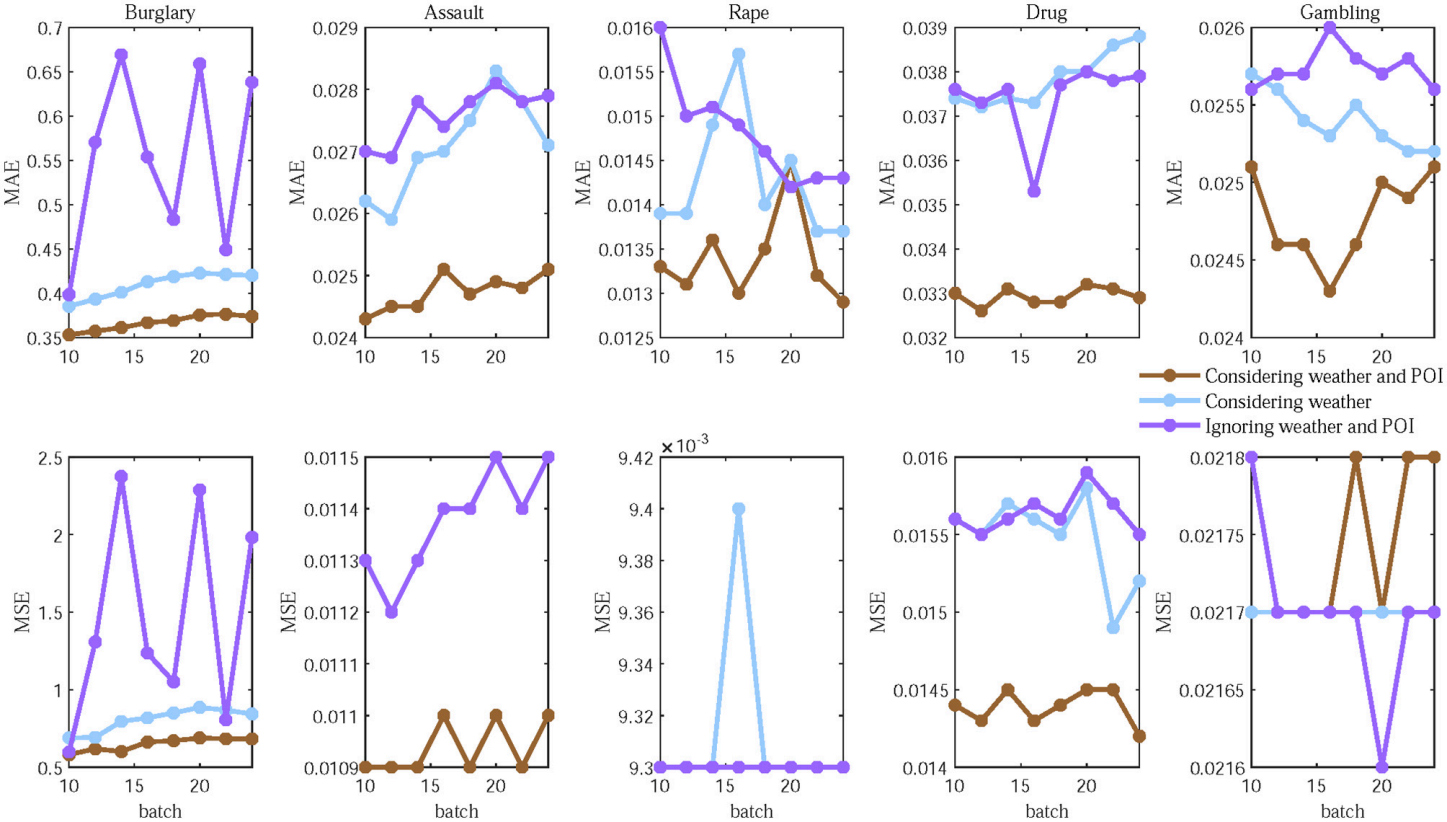
Prediction performance of different types of crimes varies with the size of the batch.

Effective feature selection has a significant impact on the performance of the spatio-temporal crime prediction model. To verify the improvement in prediction performance brought by weather and POI factors, and whether the proposed model can effectively perceive these two factors, we conducted an ablation study. To isolate the contribution of environmental factors, we compared three model configurations: a baseline excluding both weather and POI; an intermediate model adding weather features; and our full model integrating both. This sequential ablation cleanly quantifies the marginal gain from dynamic weather signals and the additional benefit of static POI context. To better illustrate the model's ability to perceive weather and POI factors, we present in Table 3 and Figure 5 the prediction results of different crime types across a range of batch sizes. This additional dimension of analysis allows us to verify that the observed influence of environmental features is consistent and not an artifact of a specific training batch configuration. Overall, the full model (with both features) outperforms the two ablated variants, demonstrating that our model effectively captures the relationships between crime and both weather and POI distributions. Specifically, we make the following observations.

(1) For burglary and assault, the fluctuations in MAE and MSE are substantial when ignoring POI and weather features. After considering weather features, MAE and MSE show significant improvements, especially for burglary. Furthermore, the overall prediction trend of the model becomes more stable when both weather and POI features are incorporated, resulting in MAE and MSE that are significantly lower than in the other two scenarios. This indicates that weather and POI have a substantial impact on burglary and assault, and our model effectively captures the relationship between these factors and the changing patterns of these two crimes.
(2) For burglary, drugs, and assault, the values of MAE and MSE remain relatively stable after incorporating weather features, but the improvement in crime prediction performance is not very significant compared to the scenario that ignores weather and POI. Fortunately, a notable improvement is observed when adding POI distribution data alongside weather features, especially for drugs. However, the MSE for gambling shows very slight fluctuations, mainly attributed to gambling's high concealment, which leads to variable reported data and a relatively low sample size that amplifies random training noise—this level of fluctuation is negligible and can be disregarded. This suggests that burglary, drugs, and gambling differ from other crime types, as they exhibit stronger correlations with specific population distributions influenced by psychological motives and social contexts, while being less sensitive to weather factors (Tipping et al., 2025).

**Table 3:** Prediction results of different types of crimes under different batch sizes.

**Batch size**	**Considering weather and POI**									
	**Burglary**		**Assault**		**Rape**		**Drugs**		**Gambling**	
	**MAE**	**MSE**	**MAE**	**MSE**	**MAE**	**MSE**	**MAE**	**MSE**	**MAE**	**MSE**
10	0.3530	0.5812	0.0243	0.0109	0.0133	0.0093	0.0330	0.0144	0.0251	0.0217
12	0.3572	0.6187	0.0245	0.0109	0.0131	0.0093	0.0326	0.0143	0.0246	0.0217
14	0.3611	0.6009	0.0245	0.0109	0.0136	0.0093	0.0331	0.0145	0.0246	0.0217
16	0.3668	0.6629	0.0251	0.0110	0.0130	0.0093	0.0328	0.0143	0.0243	0.0217
18	0.3691	0.6703	0.0247	0.0109	0.0135	0.0093	0.0328	0.0144	0.0246	0.0218
20	0.3755	0.6883	0.0249	0.0110	0.0145	0.0093	0.0332	0.0145	0.0250	0.0217
22	0.3763	0.6833	0.0248	0.0109	0.0132	0.0093	0.0331	0.0145	0.0249	0.0218
24	0.3739	0.6823	0.0251	0.0110	0.0129	0.0093	0.0329	0.0142	0.0251	0.0218
**Batch size**	**Considering weather**									
10	0.3853	0.6893	0.0262	0.0111	0.0139	0.0093	0.0374	0.0156	0.0257	0.0217
12	0.3933	0.6920	0.0259	0.0111	0.0139	0.0093	0.0372	0.0155	0.0256	0.0217
14	0.4010	0.7953	0.0269	0.0113	0.0149	0.0093	0.0374	0.0157	0.0254	0.0217
16	0.4130	0.8182	0.0270	0.0113	0.0157	0.0094	0.0373	0.0156	0.0253	0.0217
18	0.4187	0.8501	0.0275	0.0114	0.0140	0.0093	0.0380	0.0155	0.0255	0.0217
20	0.4227	0.8854	0.0283	0.0114	0.0145	0.0093	0.0380	0.0158	0.0253	0.0217
22	0.4213	0.8678	0.0278	0.0114	0.0137	0.0093	0.0386	0.0149	0.0252	0.0217
24	0.4201	0.8434	0.0271	0.0108	0.0137	0.0093	0.0388	0.0152	0.0252	0.0217
**Batch size**	**Ignoring weather and POI**									
10	0.3984	0.5980	0.0270	0.0113	0.0160	0.0093	0.0376	0.0156	0.0256	0.0218
12	0.5704	1.3079	0.0269	0.0112	0.0150	0.0093	0.0373	0.0155	0.0257	0.0217
14	0.6692	2.3740	0.0278	0.0113	0.0151	0.0093	0.0376	0.0156	0.0257	0.0217
16	0.5536	1.2358	0.0274	0.0114	0.0149	0.0093	0.0353	0.0157	0.0260	0.0217
18	0.4832	1.0501	0.0278	0.0114	0.0146	0.0093	0.0377	0.0156	0.0258	0.0217
20	0.6588	2.2882	0.0281	0.0115	0.0142	0.0093	0.0380	0.0159	0.0257	0.0216
22	0.4490	0.8053	0.0278	0.0114	0.0143	0.0093	0.0378	0.0157	0.0258	0.0217
24	0.6380	1.9824	0.0279	0.0115	0.0143	0.0093	0.0379	0.0155	0.0256	0.0217

### 5.5 Performance comparison for crime prediction

Table 4 shows the MSE and MAE of all comparative methods, from which it can be observed that our model significantly outperforms all others in predicting all types of crimes. Notably, the model achieves more pronounced performance gains for crime types with higher sparsity (e.g., rape with 0.09 records per grid per day and gambling with 0.17 records), verifying its effectiveness in mitigating data sparsity. We attribute these improvements to the following factors:

(1) By employing transfer learning, the knowledge gained from other crime distributions can be used to predict a specific type of crime, facilitating feature sharing across different types of crimes.
(2) Adaptive weighting enables the model to learn effectively from different types of crime distributions, enhancing its flexibility and robustness against the data sparsity of individual crime types. This mechanism allows the proposed CATL model to adjust the importance of different types of crimes during the training process, thereby improving performance and robustness in predicting various types crime.
(3) For crime types with relatively low incidence rates, such as gambling, the use of transfer learning can effectively enhance the model's robustness, which is often compromised by data sparsity. By leveraging knowledge from other types of crimes with sufficient data, the CATL model can better generalize and make more accurate predictions for sparse-data crime categories.

**Table 4:** Comparison between different models in terms of MAE and MSE.

**Model**	**Crime category**									
	**Burglary**		**Assault**		**Rape**		**Drugs**		**Gambling**	
	**MAE**	**MSE**	**MAE**	**MSE**	**MAE**	**MSE**	**MAE**	**MSE**	**MAE**	**MSE**
TGCN	0.8008	1.0902	0.0477	0.0116	0.0172	0.0096	0.0576	0.0166	0.0444	0.0222
DCRNN	0.8788	1.2071	0.0376	0.0112	0.0206	0.0096	0.0528	0.0163	0.0366	0.0221
STGCN	0.7433	1.8910	0.0279	**0.0108**	0.0172	0.0095	0.0505	0.0173	0.0364	0.0223
LSTM	0.5599	1.2433	0.0677	0.0142	0.0481	0.0104	0.0932	0.0249	0.0480	0.0220
GRU	0.6323	0.9946	0.0569	0.0131	0.0745	0.0133	0.0697	0.0232	0.0494	0.0228
ConvLSTM	0.4231	0.8869	0.0388	0.0137	0.0167	**0.0093**	0.0544	0.0233	0.0298	0.0226
GConvLSTM	0.8593	1.2329	0.0364	0.0112	0.0200	0.0096	0.0492	0.0161	0.0371	0.0221
PDFormer	0.5132	0.7931	0.0361	0.0112	0.0147	0.0083	0.0399	0.0149	0.0273	0.0220
Our Model	**0.3767**	**0.6901**	**0.0242**	**0.0108**	**0.0129**	**0.0093**	**0.0321**	**0.0141**	**0.0244**	**0.0217**

Bold values denote the minimum MAE and MSE for each crime category, indicating the best predictive performance among the compared models.

These results demonstrate that the proposed transfer learning mechanism effectively leverages shared patterns across crime types to improve prediction robustness within the studied urban context, especially for categories with sparse records.

## 6 Conclusion

Spatio-temporal crime prediction differs from traditional spatio-temporal predictions of traffic flow, temperature, and air quality due to the challenge of data sparsity. Considering the co-occurrence phenomenon among different types of crime, an adaptive transfer learning training approach is adopted to fully leverage crime data and mitigate data sparsity. The proposed approach facilitates feature sharing among different types of crime, enabling a comprehensive exploration of underlying relationships. This enhances the model's ability to recognize potential cross-type spatio-temporal correlations and improves its prediction performance and robustness when dealing with sparse and heterogeneous crime data. In practical applications, the proposed model can help police departments implement more targeted patrol strategies—for example, by allocating more resources to high-risk regions predicted for sparse crime types (e.g., rape and gambling) that are often overlooked, thereby improving the overall efficiency of urban safety management. In summary, we can draw the following conclusions:

(1) The study introduces a transfer learning framework based on ConvLSTM. When predicting a specific type of crime, this framework extracts features from other types of crimes and incorporates them into the training process. To balance the model's attention toward distribution discrepancy losses across different types of crimes, an adaptive weight updating method that utilizes the rate of change of distribution discrepancy losses is employed. Experimental results show that the proposed approach can extract underlying relationships among different types of crime and enhance the model's prediction performance. Ablation results confirm Transfer Learning's critical role in alleviating data sparsity—removing it increases MAE by 29.6% for rape and 22.1% for gambling compared to the full model. Removing adaptive weight updating further leads to 11.9–13.6% higher MAE than the full model, as it fails to balance learning across crime types with varying sample sizes.
(2) The surrounding environment can influence the distribution of crimes. Building upon the consideration of meteorological data, we introduced the distribution of POIs and other features (e.g., population data) and analyzed the model's ability to perceive these features. Experimental results show that compared to the scenario that only considers weather features, the predictive performance for burglary, gambling, and drug crimes significantly improves when incorporating POI and population data. This further confirms the substantial impact of POI and other features on these specific types of crimes. POI features drive more pronounced improvements than weather—drug crimes show a 14.5% lower MAE when adding POIs to weather, while assault and burglary benefit more from weather, with an 8.3–10.1% MAE reduction vs. no environmental features, reflecting their different correlations with human activity and mobility.
(3) Compared to traditional spatio-temporal prediction models, our proposed approach achieves superior predictive performance across all crime types and effectively captures features among different types of crimes. More importantly, it demonstrates a robust capability to handle the dual challenge inherent to urban crime prediction: leveraging sparse data through cross-type knowledge transfer and integrating heterogeneous urban and environmental features to uncover complex, crime-specific patterns.
(4) While the proposed CATL model demonstrates effectiveness in mitigating data sparsity within the studied urban environment, this study has limitations that suggest directions for future work. Our evaluation was conducted on data from a single city and at a specific granularity. Thus, while the model shows strong robustness in handling intra-city data sparsity and cross-type correlations, its generalizability to cities with different geographic, demographic, and crime patterns requires further validation.

Future research will focus on three specific directions: first, cross-city evaluation using datasets from cities with varying geographic conditions such as coastal areas, inland regions, and diverse topographies, as well as demographic characteristics including differing population densities to verify generalizability; second, testing on finer-grained data such as hourly intervals or 1 km × 1 km grids while developing targeted strategies to address extreme sparsity; and third, integrating domain knowledge from criminal psychology and urban planning into the transfer learning framework to further enhance prediction accuracy.

## Data Availability

The raw data supporting the conclusions of this article will be made available by the authors, without undue reservation.
